# Uncovering Synergistic Mechanism of Chinese Herbal Medicine in the Treatment of Atrial Fibrillation with Obstructive Sleep Apnea Hypopnea Syndrome by Network Pharmacology

**DOI:** 10.1155/2019/8691608

**Published:** 2019-12-23

**Authors:** Ting Mao, Jingchun Zhang, Yu Qiao, Bei Liu, Shan Zhang

**Affiliations:** ^1^Cardiovascular Diseases Center of Xiyuan Hospital, China Academy of Chinese Medical Sciences, Beijing 100091, China; ^2^Institute of Cardiovascular Diseases, China Academy of Chinese Medical Sciences, Beijing 100091, China

## Abstract

Paroxysmal atrial fibrillation (AF) combined with obstructive sleep apnea hypopnea syndrome (OSAHS) is very common in clinical practice. Traditional Chinese medicine (TCM) rule of regulating the liver based on psycho-cardiology shows satisfactory effectiveness in the treatment of paroxysmal AF combined with OSAHS. However, its underlying pharmacological mechanism has not yet been elucidated. This study applied network pharmacology to identify 94 active components in the six TCM liver-regulating herbs and 182 corresponding targets from several databases and comprehensive literature studies, as well as retrieved AF combined with OSAHS-related targets. Cytoscape software was adopted to construct the component-component target network and component-putative target-AF combined with OSAHS target network. Then, we obtained 38 putative therapeutic targets against AF combined with OSAHS. After the production of a putative therapeutic target interaction network, topological analysis was adopted to determine the core targets of TCM liver-regulating herbs in the treatment of paroxysmal AF combined with OSAHS. For all putative therapeutic targets, biological process analysis and pathway enrichment analysis were utilized to investigate the possible mechanism of TCM liver-regulating herbs in the treatment of paroxysmal AF combined with OSAHS. Mechanistically, it included positive regulation of nitric oxide biosynthetic process, aging, response to hypoxia, TNF signaling pathway, HIF-1 signaling pathway, PI3K-Akt signaling pathway, neuroactive ligand-receptor interaction, and calcium signaling pathway. Especially, six core targets of TCM liver-regulating herbs, namely, TNF, STAT3, AKT1, IL-6, TP53, and INS, were significant in the regulation of the above biological processes and pathways. This study demonstrates the multicomponent, multitarget, and multipathway feature of TCM liver-regulating herbs, provides an extensional foundation for further research, and facilitates the reasonable application of TCM liver-regulating herbs in treating paroxysmal AF combined with OSAHS.

## 1. Introduction

Atrial fibrillation (AF) is one of the most common types of arrhythmia during clinical practice and is associated with elevated cardiovascular morbidity and mortality rate [1]. Obstructive sleep apnea hypopnea syndrome (OSAHS) is an independent and vital risk factor for AF [2]. Several studies have revealed the increased incidence of AF in patients with OSAHS [3–6]. Conventional ventilator therapy is not recommended for moderate OSAHS patients. Antiarrhythmia medicine is classical therapeutic treatment for AF, but adverse effects discouraged the patient compliance. Therefore, it is recognized that there is still an urgent need for more effective and safer treatment for AF combined with OSAHS. The latest research shows that Chinese herbal medicine can improve physical fitness, increase the ability to resist hypoxia, and inhibit inflammatory response, implying the possibility of integrating Traditional Chinese medicine (TCM) to improve paroxysmal AF combined with OSAHS and its prognosis [7]. Therefore, Chinese herbal medicine alone or in combination with other drugs may become a promising drug for the treatment of AF combined with OSAHS.

TCM characteristic of AF patients with OSAHS who frequently showed irritability, anxiety, and depression is more likely complicated with qi depression and stagnation [8]. TCM rule of regulating the liver based on psycho-cardiology combined with tonifying qi to treat paroxysmal AF has comprehensive effectiveness in clinical practice for many years. Our previous analysis showed Chinese herbal medicine including Ziziphi Spinosae Semen (Suan Zao Ren (SZR)), Nardostachys Chinensis Batal (Gan Song (GS)), Corydalis Rhizoma (Yan Hu Suo (YHS)), Agrimony (Xian He Cao (XHC)), Tribulus terrestris (Ji Li (JL)), and Concha Ostreae (Mu Li (ML)) were more frequently used in related diseases [9]. Additionally, the clinical retrospective survey results conducted by our research team confirmed that these six TCM liver-regulating herbs are effective in relieving clinical symptoms of palpitation, maintaining sinus rhythm, shortening the duration of AF episodes, and improving the quality of life [9]. It is reported that Nardostachys Chinensis Batal extract has a membrane stabilizing effect, and it has a concentration-dependent inhibition on the sodium channel and L-type calcium channel in rabbit single ventricular myocytes [10]. Moreover, the classical compound Shensong Yangxin capsule containing Nardostachys Chinensis Batal is recommended to treat paroxysmal AF according to guidelines for the treatment of AF in China. Therefore, these TCM liver-regulating herbs have the potential to treat AF combined with OSAHS. Additionally, studies have found that Corydalis Rhizoma, Tribulus terrestris, and Ziziphi Spinosae Semen may maintain cellular homeostasis through regulating the level of gene expression, thereby improving overall function of cardiomyocytes. For instance, the antiarrhythmic effect of Corydalis Rhizoma is mainly through inhibition of IK, IK1, and tail current, prolonging the action potential duration and effective refractory period as well as suppressing calcium current and alleviating calcium overload [11]. Tribulus terrestris can protect cardiomyocyte from damage induced by adriamycin, which is possibly involved with its effect of resisting oxygen-free radical [12]. The research suggested that Jujuboside A may protect against norepinephrine-induced apoptosis of cardiomyocytes via modulation of the MAPK and AKT signaling pathways [13]. However, the feature of these above conventional experimental studies is one-drug-one-target-one-pathway, which ignores the multicomponent, multitarget, and multipathway characteristic of herbal formulae. Therefore, its active components and underlying pharmacological mechanism remain to be elucidated completely.

**Figure 1:**
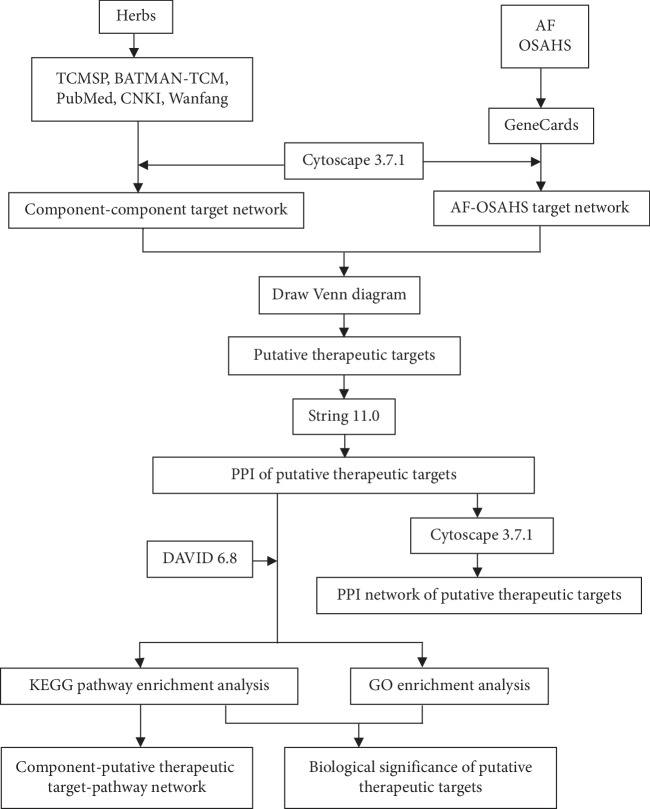
Technology roadmap of the pharmacological mechanism of TCM liver-regulating herbs in treating paroxysmal AF combined with OSAHS through network pharmacology.

Additionally, herbal formulae possess the advantages of simplicity, convenience, efficiency, and economy. TCM syndrome differentiation and treatment conforms to the concept of individualized therapy in modern medicine. Herbal formulae can exert its therapeutic effect through acting on multiple targets and multiple pathways, so it is difficult to elucidate the mechanisms of action through conventional research [14]. Fortunately, with the rapid development and widespread application of bioinformatics, the network pharmacology approach can be utilized to systemically reflect the correlation among multiple components, multiple targets, multiple pathways, and complex diseases. Therefore, the study aims to elucidate the pharmacological mechanism of TCM liver-regulating herbs in treating paroxysmal AF combined with OSAHS through network pharmacology. The technology roadmap is described in Figure 1.

## 2. Materials and Methods

### 2.1. Identification of Active Components

Active components of six TCM liver-regulating herbs were collected both from the Traditional Chinese medicine systems pharmacology (TCMSP ver.2.3, http://lsp.nwu.edu.cn/tcmsp.php, updated on May. 31, 2014) database and Bioinformatics Analysis Tool for Molecular mechANism of Traditional Chinese Medicine (BATMAN-TCM) platform (http://bionet.ncpsb.org/batman-tcm/). TCMSP database is the largest pharmacological data platforms for TCM. They encompass many herbs, chemical components, and pharmacokinetic properties, which include absorption, distribution, metabolism, and excretion (ADME) information in the Pharmacopoeia of the People's Republic of China (2010 edition). Owing to the disadvantages of biological experiments as being time-consuming and expensive, identification of ADME (absorption, distribution, metabolism, and excretion) characteristic has now become an acknowledged model in pharmaceutical research. In this study, two important ADME parameters including bioavailability (OB) and drug-likeness (DL) were utilized to screen bioactive components. The screening criteria are OB ≥ 30% and DL ≥ 0.18 [15, 16]. In addition, the databases of the China National Knowledge Infrastructure, Wanfang, and PubMed were also used to supplement any other omitted components. The cutoff parameter was set at 20, and the adjusted *P* value cutoff was set at 0.05 when we retrieved active components in BATMAN-TCM platform.

### 2.2. Potential Targets of Active Components

The validated targets of the active components were retrieved from both TCMSP database and BATMAN-TCM platform. BATMAN-TCM platform was used to predict targets of Concha Ostreae. Its principle is to identify potential target by calculating drug similarity and comparing target interactions [17]. The cutoff parameter was set at 20, and the adjusted *P* value cutoff was set at 0.05. Then, we used UniProt Knowledgebase (UniProtKB, http://www.uniprot.org/) to convert target protein names of active components into standard target gene names. This database provides the scientific community with a comprehensive, high-quality, and freely accessible resource of protein sequence and functional information. The target protein names were entered into UniProtKB, with the organism limited into “*Homo sapiens*,” prior to the retrieval of the official symbol. Finally, the validated and predicted targets of the active components from six TCM liver-regulating herbs were obtained.

### 2.3. Targets Related to AF Combined with OSAHS

*GeneCards* (http://www.genecards.org/) database is a searchable, integrative database that provides comprehensive, user-friendly information on all annotated and predicted human genes. It automatically integrates gene-centric data from 150 network sources, including genomic, transcriptomic, proteomic, genetic, clinical, and functional information. Then, targets were predicted by identifying the target genes associated with AF combined with OSAHS through GeneCards database.

### 2.4. Protein-Protein Interaction Data

Putative targets with therapeutic effects against AF combined with OSAHS were identified by intersecting the component targets and disease targets. Draw Venn Diagram (http://bioinformatics.psb.ugent.be/webtools/Venn/) was applied online to acquire the intersection between component targets and AF combined with OSAHS-related targets. The common target proteins of component and disease were used to construct protein-protein interaction (PPI) network on the Search Tool for the Retrieval of Interacting Genes/Proteins (STRING, ver. 11.0, https://string-db.org/) platform, which is a database of validated and predicted PPIs, including both direct and indirect interactions among related proteins [18]. We set the organism limited to “*Homo sapiens*.” Eventually, we identified the PPI data of significant targets by the combined score. The combined score represents the interaction confidence of the protein, which has a positive correlation [19].

### 2.5. Network Construction

Cytoscape 3.7.1 (http://cytoscape.org/) software was adopted to visually display and analyze the network structure of closely related proteins [20]. It can visualize molecular interaction networks, biological pathways, and related genes. Cytoscape renders a set of data integration, analysis, and visualization functions to analyze complicated networks. We input targets and PPI data into Cytoscape to construct networks. There, three networks were constructed in this study as follows: (1) a component-component target network was established by joining active components of herbs and corresponding targets; (2) a component-putative target-AF combined with OSAHS target network was constructed by intersecting the component-component target network and AF combined with OSAHS-related target network; and (3) a component-putative therapeutic target-pathway network was constructed by linking herb components, potential therapeutic targets, and pathways. The core network was determined by topological analysis. We applied network topological feature value to collect core network. Three topological feature parameters of each node were evaluated in the network. The three parameters contain degree centrality (DC), betweenness centrality (BC), and closeness centrality (CC) of nodes. The DC represents the number of edges connecting two nodes in the network [21]. The higher the degree is, the more important the node is. The BC shows the participation of nodes in the shortest part of the network and embodies the ability of nodes to handle the rate of information flow in the network [22]. The CC is the inverse of the sum of the distance from a node to other nodes. The magnitude of the important parameters is positive proportional to the importance of node in the network [23]. Firstly, we selected “DC > 2 × median” as the primary screening criteria. Eventually, nodes that satisfy the median of aforementioned topological parameters simultaneously is called core targets, and the related network is regarded as core network.

### 2.6. Gene Ontology and Pathway Enrichment Analysis

The Database for Annotation, Visualization, and Integrated Discovery (DAVID 6.8, https://david.ncifcrf.gov/) was utilized for the Gene Ontology (GO) and Kyoto encyclopedia of genes and genomes (KEGG) enrichment analysis, which are the classical analysis approach performed to elucidate the properties of candidate targets in gene function and pathway [24, 25]. GO enrichment analysis involves biological process (BP), cell component (CC), and molecular functions (MF). DAVID provides high-throughput functional annotation bioinformatics to perform functional annotation and enrichment analysis. The cutoff of recognized GO terms is set to FDR < 0.01 and KEGG pathways is set to *P* value <0.05 [26, 27].

## 3. Results

### 3.1. Herbs Active Component Data

We obtained 265 components from the six TCM liver-regulating herbs after removing duplication. The bioactive components were screened out by OB and DL. The 94 active components from the six TCM liver-regulating herbs were searched in domestic and foreign literature databases, TCMSP database, and BATMAN-TCM platform. In addition to the above 86 bioactive components, 8 components with low OB and DL were considered active components, because of their reported therapeutic effects. These components were also selected for further study because of their good pharmacological effects. In brief, 94 active components from the six TCM liver-regulating herbs were collected for further research analysis, including 9 in SZR, 8 in GS, 51 in YHS, 10 in XHC, 14 in JL, and 6 in ML (Supplemental [Supplementary-material supplementary-material-1]).

### 3.2. Target Prediction of Herb Component

A total of 2152 candidate targets from the 94 active components were identified by applying the target prediction model. Then, these targets were standardized into official gene symbol. 2071 official gene symbols were finally transformed. The amount of potential target genes was 80, 90, 1493, 232, 143, and 33 from SZR, GS, YHS, XHC, JL and ML, respectively. Eventually, we identified 182 target genes from 2071 potential target genes hit by 94 active components after deletion of duplication (Supplemental [Supplementary-material supplementary-material-1]). Targets from SZR, GS, and YHS were overlapped significantly with YHS, which indicates that different components from herbs can share the same or similar targets. At the same time, it indicated that targets from YHS play a vital role in synergistic effects.

### 3.3. Component-Component Target Network Analysis

**Figure 2:**
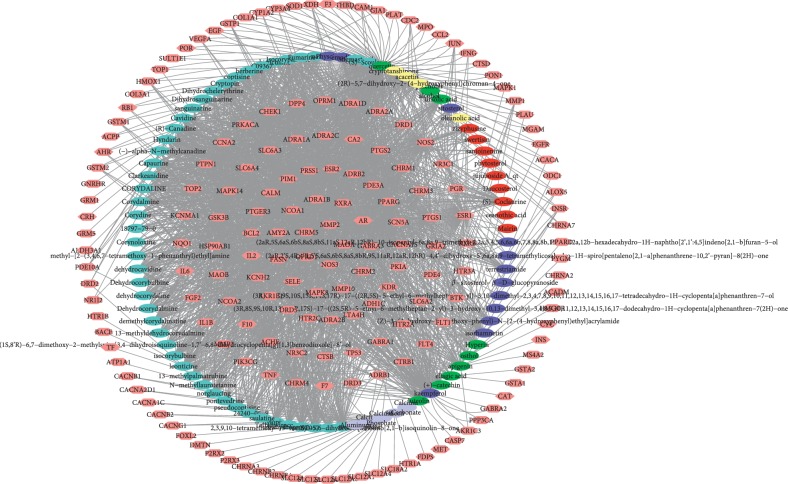
Active component-component target network. Pink represents the component target. Red, yellow, light blue, green, dark blue, and purple represent active components of SZR, GS, YHS, XHC, JL, and ML, respectively.

To further illustrate the action of components on potential targets, we applied Cytoscape 3.7.1 software to visualize the component target network. The network consists of 268 nodes (94 active components and 182 component targets) and 2156 edges (Figure 2). The network suggests that not only a single component acts on multiple targets but also various targets are hit by one component. Specially, the number of candidate targets for quercetin, kaempferol, and isocorypalmine are 164, 104, and 50, respectively, indicating that these three active components are pivotal in these TCM liver-regulating herbs. This implies that the herb component may play a pharmacological role in other diseases in addition to AF. Meanwhile, the network illustrates the multicomponent, multitarget, and multidisease characteristics of herbal formulae.

### 3.4. Component-Putative Target-AF Combined with OSAHS Target Network Analysis

**Figure 3:**
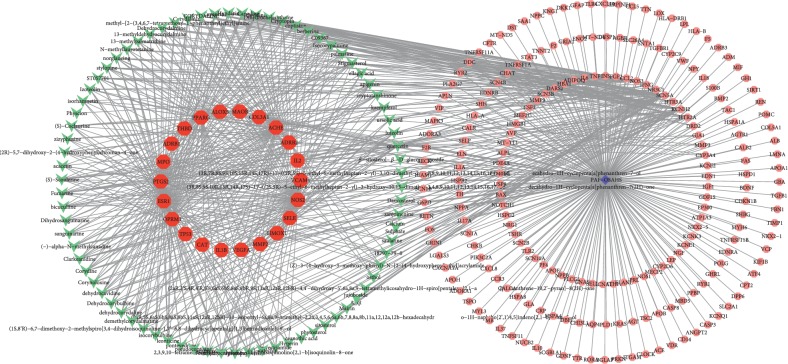
Component-putative target-AF combined with OSAHS target network. The green nodes represent component, the red nodes represent common targets between component targets and AF combined with OSAHS targets, and pink nodes represent AF combined with OSAHS targets.

We retrieved 232 targets of AF combined with OSAHS (Supplemental [Supplementary-material supplementary-material-1]). TCM liver-regulating herbs shared 50 common targets with AF, while 75 with OSAHS. There are 38 overlapping targets of herbs in treating AF combined with OSAHS. We constructed a network to illustrate the relationship among herb components, component targets with therapeutic effects against AF combined with OSAHS, and AF combined with OSAHS-related targets. The network consists of 311 nodes (78 component nodes, 38 common target nodes between component targets and AF combined with OSAHS-related targets, 232 AF combined with OSAHS-related target nodes, and 1 AF combined with OSAHS node) and 802 edges (Figure 3). This network shows that the component targets are also shared by AF combined with OSAHS targets, which suggests that herbs may indirectly regulate disease-related proteins. At least, these six herbs may also indirectly affect related targets by regulating common proteins.

### 3.5. PPI Network Analysis

**Figure 4:**
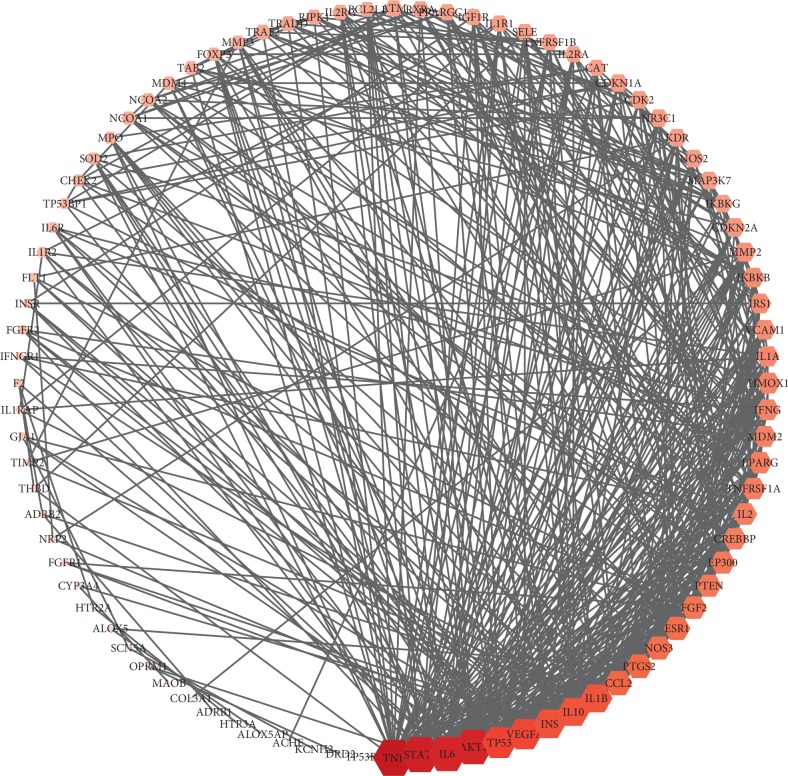
PPI network of 38 putative therapeutic targets of herbs in the treatment of AF combined with OSAHS. The nodes represent common targets for herb treatment of AF combined with OSAHS as well as other related human target genes. The size of nodes suggests the degree, and the larger the nodes are and closer the nodes are to red, the higher the degree they have.

In order to elucidate the systemic and pharmacological mechanism of TCM liver-regulating herbs in treating AF combined with OSAHS, we first constructed and visualized a PPI network of 38 putative targets with therapeutic effects against AF combined with OSAHS. Totally, we obtained 88 nodes and 505 edges in this network (Figure 4). Then, topological analysis was adopted to determine the core targets of TCM liver-regulating herbs in the treatment of paroxysmal AF combined with OSAHS. For instance, we first applied the topological method to evaluate the hub network according to the topology analysis screening criteria “DC ≥ 2 × median.” On this basis, the target that satisfied the median of DC, BC, and CC synchronously is screened as the core targets. The PPI network is beneficial to explore the interaction of various proteins in complex diseases. Finally, we identified six core targets of TCM liver-regulating herbs in treating AF combined with OSAHS (Supplemental [Supplementary-material supplementary-material-1]). These core targets included TNF, STAT3, AKT1, IL6, TP53, and INS, indicating that they serve as vital targets of TCM liver-regulating herbs in treating AF combined with OSAHS.

### 3.6. GO and KEGG Enrichment Analysis

**Figure 5:**
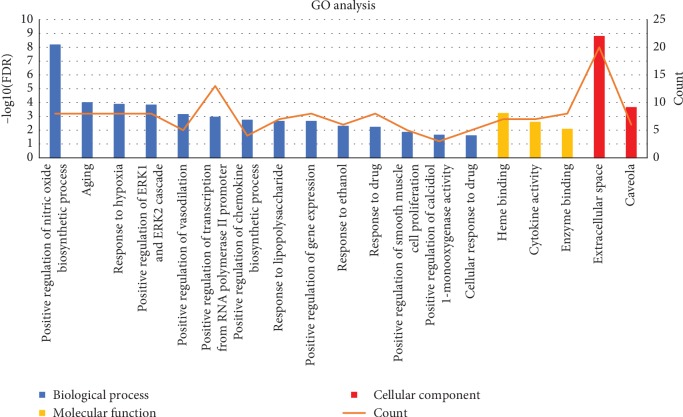
GO enrichment analysis of putative therapeutic targets. The *Y*-axis shows the enrichment scores of these terms or the counts of targets, and the *X*-axis shows significantly enriched GO categories of the target genes (FDR < 0.01).

To illustrate the mechanism of herbs on AF combined with OSAHS from a systematic level, we performed GO analysis and KEGG pathway enrichment analysis for 38 putative therapeutic targets. Totally, we identified 233 enrichment results in the related items of biological process, 41 of molecular functions, and 23 of cell components (Supplemental [Supplementary-material supplementary-material-1]). Then, 19 significant enriched GO terms were determined, including 14 biological processes, 3 molecular functions, and 2 cell components (Figure 5). For biological process, these putative therapeutic targets were mainly enriched in positive regulation of nitric oxide biosynthetic process (GO:0045429), aging (GO:0007568), response to hypoxia (GO:0001666), positive regulation of ERK1 and ERK2 cascade (GO:0070374), positive regulation of vasodilation (GO:0045909), positive regulation of transcription from RNA polymerase II promoter (GO:0045944), positive regulation of chemokine biosynthetic process (GO:0045080), response to lipopolysaccharide (GO:0032496), positive regulation of gene expression (GO:0010628), response to ethanol (GO:0045471), response to drug (GO:0042493), positive regulation of smooth muscle cell proliferation (GO:0048661), positive regulation of calcidiol 1-monooxygenase activity (GO:0060559), and cellular response to drug (GO:0035690). For molecular function, the targets were enriched in heme binding (GO:0020037), cytokine activity (GO:0005125), and enzyme binding (GO:0019899). For the cell component, the targets were enriched in extracellular space (GO:0005615) and caveola (GO:0005901). Therefore, the results indicated that TCM liver-regulating herbs mainly exert therapeutic effects by regulating these biological processes, molecular functions, and cell components.

**Figure 6:**
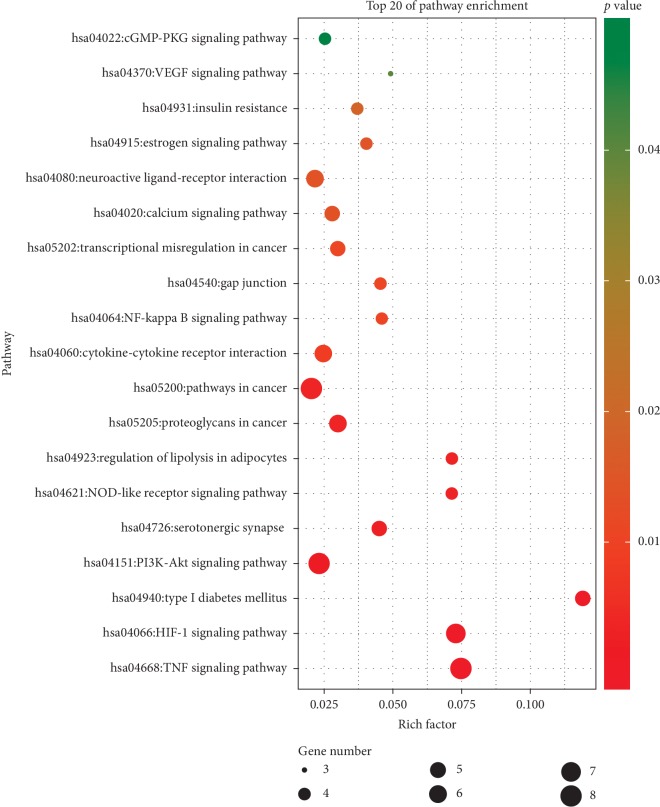
KEGG pathway enrichment analysis of putative therapeutic targets. The *Y*-axis represents significantly pathways of the target genes, and the *X*-axis shows the rich factor. The rich factor represents the ratio of the number of target genes belonging to a pathway to the number of all the annotated genes located in the pathway. A higher rich factor represents a higher level of enrichment. The color of the dot corresponds to different *P* values, and the size of the dot reflects the number of target genes expressed in the pathway.

We performed KEGG pathway enrichment analysis to expound the pathways among the 38 putative therapeutic targets of herbs in the treatment of AF combined with OSAHS. A total of 48 pathways were obtained (Supplemental [Supplementary-material supplementary-material-1]), 19 of which were significantly enriched (Figure 6), mainly including TNF signaling pathway (hsa04668), HIF-1 signaling pathway (hsa04066), cytokine-cytokine receptor interaction (hsa04060), PI3K-Akt signaling pathway (hsa04151), neuroactive ligand-receptor interaction (hsa04080), serotonergic synapse (hsa04726), and calcium signaling pathway (hsa04020). It indicated that these top 7 signaling pathways might be involved in the critical pharmacological mechanism of TCM liver-regulating herbs in the treatment of AF combined with OSAHS. It is precisely these above transduction pathways that play essential role in OSAHS to induce and aggravate AF. Meanwhile, it could provide potential evidence for exploring drug therapeutic targets for AF combined with OSAHS.

### 3.7. Component-Putative Therapeutic Target-Pathway Network Analysis

**Figure 7:**
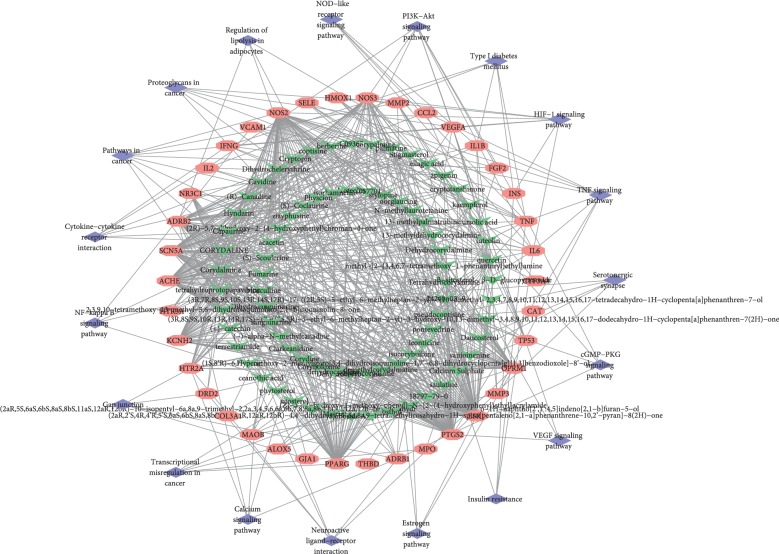
Putative therapeutic active component-putative therapeutic target and pathway network. Green represents the putative therapeutic active component, pink represents the putative therapeutic target, and purple represents the KEGG pathway.

In order to more holistically present the mechanism of TCM liver-regulating herbs in treating AF combined with OSAHS, we intersected component-component target network, AF combined with OSAHS network, and the involved KEGG pathway to construct a component-putative therapeutic target-pathway network (Figure 7). The interaction above revealed the pharmacological and molecular mechanisms of TCM liver-regulating herbs treating AF combined with OSAHS.

## 4. Discussion

Chinese herbal medicine with multiple components produces therapeutic effect through acting on multiple targets and pathways. Network pharmacology is a comprehensive approach based on traditional pharmacology, bioinformatics, chemoinformatics, and network biology. This approach enables researchers to explore regulation of the signaling pathways with multiple targets and optimize the objective of clinical trials [28]. It is widely considered as a supplementary approach to identify components and pharmacological mechanisms of Chinese herbal medicine from a comprehensive perspective [29]. Therefore, we apply network pharmacology to construct a component-component target network, component-putative therapeutic target-AF combined with OSAHS target network, and component-putative therapeutic target-pathway network to systematically explore the pharmacological and molecular mechanisms of TCM liver-regulating herbs in the treatment of paroxysmal AF combined with OSAHS.

In the component-component target network, quercetin, kaempferol, and sitosterol were recognized as significant active components. Quercetin and kaempferol account for most of the putative targets from six TCM liver-regulating herbs, suggesting that the two active components play critical role in pharmacological mechanism of TCM liver-regulating herbs. Quercetin was shown to be associated with a variety of AF combined with OSAHS targets, including TNF, IL-6, SCN5A, NOS2, NOS3, and PPARG. Several reports have demonstrated that quercetin possesses antioxidative, anti-inflammatory activities, antiproliferative activities, and free radical-scavenging [30, 31]. It is reported that quercetin prevented cardiac hypertrophy by proteasome inhibition and activation of GSK-3*α*/*β*, which is related to upstream (AKT, LKB1/AMPK*α*) and downstream hypertrophic factors, such as ERK, histone H3, *β*-catenin, and GATA4 [32]. Early research demonstrated that quercetin decreased myocardial [Ca^2+^]_i_-oscillation frequency and prevented cardiac remodeling [33]. The investigation indicated that treatment with kaempferol protects against cardiac hypertrophy, and its cardioprotective effect may be partially explained by the inhibition of the ASK1/MAPK signaling pathway and the regulation of oxidative stress [34]. Additionally, kaempferol attenuated cardiac fibroblast inflammation through inhibition of release of TNF-*α*, IL-1*β*, IL-6, and IL-18 and suppression of activation of NF-*κ*B and AKT [35]. The proliferation and activation of cardiac fibroblasts can be inhibited by kaempferol [36].

In the AF combined with the OSAHS target network, INS, IL6, and TNF are selected as the hub nodes. Insulin resistance induces both atrial structural remodeling and abnormal intracellular calcium homeostasis, contributing to increased AF susceptibility [37]. It is reported that insulin resistance-induced AF was associated with impairment in the trafficking and expression of the major cardiac isoform GLUT4 and the novel isoform GLUT8 [38]. Inflammatory markers IL-6 and TNF play an essential role in the occurrence and development of AF. Conversely, AF generates an inflammatory response that further enhances atrial remodeling and perpetuates the arrhythmia. Studies have reported that higher levels of IL-6 and TNF as well as elevated neutrophil and lymphocyte ratios have been reported in patients with AF compared with those in the sinus rhythm [39]. Meanwhile, AF can be prevented by inhibiting inflammation, further highlighting the relation between inflammation and AF [40].

In the component-putative therapeutic target-pathway network, 38 therapeutic targets were primarily modulated by quercetin, luteolin, kaempferol, apigenin, stigmasterol, isocorypalmine, and hyndarin. The 6 core targets were determined by network topology analysis, including TNF, STAT3, AKT1, IL-6, TP53, and INS. Various inflammatory cytokines regulate the function of ion channels and calcium homeostasis. TNF induces abnormal Ca^2+^ level and arrhythmogenesis in cardiomyocytes [41]. TNF can also decrease the expression of sarcoplasmic/endoplasmic reticulum Ca-ATPase [42]. Furthermore, mice with an elevated TNF level have increased susceptibility to AF and accelerated spontaneous AF onset [43]. These studies have suggested that TNF may be crucial for AF occurrence and atrial electrical remodeling. IL-6, levels of circulating IL-6, and other proinflammatory molecules have consistently been associated with a risk for AF and its recurrence after catheter ablation [44]. It is reported that IL-6 elevation rapidly induces atrial electrical remodeling by downregulating cardiac connexins [45]. Targeting inflammatory mediators, particularly IL-6, may represent in perspective an innovative approach in antiarrhythmic therapy [46]. For STAT3, activation of the JAK-STAT3 signaling pathway may lead to fibrosis in many organs, including the heart. Atrial fibrosis in patients with AF is induced by increase in ROS production and subsequently activation of STAT3 and SMAD3 signaling [47]. For AKT1, Ang II infusion upregulated PSMB10 expression and AKT1 activation leading to cardiac fibrosis [48]. TP53, which can control cell proliferation, DNA repair, and cell death, is one of the most commonly inactivated tumor suppressors in human cancers [49]. For INS, it is suggested that insulin resistance promotes atrium electrical remodeling and might be related to AF recurrence after PVI [50]. Insulin-resistant (IR) animals demonstrated an increased vulnerability to induced AF, as well as spontaneous AF.

In this study, we performed enrichment analysis to clarify the multiple mechanisms of TCM liver-regulating herbs against AF combined with OSAHS from a holistic and systematic perspective. In the GO enrichment analysis, the targets were closely related to positive regulation of nitric oxide biosynthetic process, aging, response to hypoxia, and positive regulation of ERK1 and ERK2 cascade. The results suggested that TCM liver-regulating herbs may exert therapeutic effect through acting on these biological processes. In the KEGG pathway enrichment analysis, we obtained that the pathways mainly associated with AF combined with OSAHS were TNF signaling pathway, HIF-1 signaling pathway, PI3K-Akt signaling pathway, neuroactive ligand-receptor interaction, and calcium signaling pathway. It is shown that oxidative stress and inflammation associated with OSAHS have been implicated in AF initiation and perpetuation [51]. Elevated oxidative stress and inflammation including HIF1 signaling pathway and TNF signaling pathway play essential role in high risk of episode of AF. Activation of systemic and atrial RAAS combining with atrial oxidative stress and inflammation may contribute to atrial remodeling in OSAHS, potentially severing as an arrhythmogenic substrate for AF occurrence [52]. Research found that telmisartan reduced susceptibility to atrial arrhythmia, ameliorated atrial remodeling, and reversed imbalances in the RAS-ERK and PI3K-Akt pathway [53]. Evidence showed that Shensong Yangxin suppressed atrial electrical remodeling by regulating autonomic nerve activity and decreased levels of TNF-*α* and IL-6 [54]. The upregulation of K^+^ channel expression and downregulation of Ca^2+^ channel expression in OSAHS experimental models contribute to shortening of AERP and increase in AF susceptibility [55]. Recurrent nocturnal apneas and hypoxia during sleep in patients with OSAHS induced sympathetic nervous system imbalance preceding the initiation of paroxysmal AF [56, 57]. Therefore, the study demonstrated that the mechanism of TCM liver-regulating herbs against AF combined with OSAHS might be closely associated with these signaling pathways.

## 5. Conclusions

In the present study, we identified 94 active components of six TCM liver-regulating herbs and 182 component targets, showing the characteristic of multicomponent and multitarget of herbs. The herbs exerted therapeutic effects on AF combined with OSAHS by acting on 38 targets, which were primarily hit by quercetin, luteolin, kaempferol, apigenin, stigmasterol, isocorypalmine, and hyndarin. Additionally, 6 core targets were identified by network topology analysis, namely, TNF, STAT3, AKT1, IL6, TP53, and INS. The GO enrichment analysis demonstrated that the therapeutic targets of herbs in the treatment of AF combined with OSAHS might be tightly associated with biological processes, for instance, positive regulation of nitric oxide biosynthetic process, aging, response to hypoxia, positive regulation of ERK1 and ERK2 cascade, positive regulation of vasodilation, and positive regulation of transcription from RNA polymerase II promoter. Moreover, the KEGG pathway enrichment analysis indicated that the action target of herbs might be closely related with multiple signaling pathway, including TNF signaling pathway, HIF-1 signaling pathway, PI3K-Akt signaling pathway, cytokine-cytokine receptor interaction, neuroactive ligand-receptor interaction, and calcium signaling pathway. These pathways play significant role in therapeutic effects of TCM liver-regulating herbs in the treatment of AF combined with OSAHS.

In summary, the present study systemically analyzed the complicate correlation among multiple components, targets, and pathways for TCM liver-regulating herbs in the treatment of AF combined with OSAHS. The results explored the pharmacological and molecular mechanisms of TCM liver-regulating herbs acting on paroxysmal AF combined with OSAHS, which may lay a good theoretical foundation for further experimental verification and facilitate the widespread application of TCM liver-regulating herbs in treating arrhythmia. However, further experimental research is urgently needed to verify the prediction.

## Data Availability

The data used to support the research are available from the Supplementary Materials uploaded with this article.

## Supplementary Materials

Supplementary Materials Supplemental Table 1: 94 active components of TCM liver-regulating herbs were collected, of which 9 in SZR, 8 in GS, 51 in YHS, 10 in XHC, 14 in JL, and 6 in ML. Supplemental Table 2: targets of the active components from TCM liver-regulating herbs. The number of these target genes was 80, 90, 1493, 232, 143, and 33 from the corresponding active components of SZR, GS, YHS, XHC, JL, and ML, respectively. After removing duplicates, we identified 182 target genes from 2071 potential target genes out of 94 bioactive components. Supplemental Table 3: we retrieved 232 targets of AF combined with OSAHS from GeneCards database. Supplemental Table 4: after the production of a putative therapeutic target interaction network, topological analysis was adopted to determine the core targets of TCM liver-regulating herbs in the treatment of paroxysmal AF combined with OSAHS. Totally, six core targets including TNF, STAT3, AKT1, IL6, TP53, and INS were identified. Supplemental Table 5: to illustrate the mechanism of herbs on AF combined with OSAHS from a systematic level, we performed GO enrichment analysis for putative therapeutic targets. Totally, we identified 233 enrichment results in the related items of biological process, 41 of molecular function, and 23 of cell component. Supplemental Table 6: KEGG pathway enrichment analysis was performed to expound the pathways among the putative therapeutic targets of herbs in the treatment of AF combined with OSAHS. A total of 48 pathways were obtained.
